# Analysis of gene expression changes in *Trichophyton rubrum* after skin interaction

**DOI:** 10.1099/jmm.0.059386-0

**Published:** 2014-05

**Authors:** Tao Liu, Xingye Xu, Wenchuan Leng, Ying Xue, Jie Dong, Qi Jin

**Affiliations:** MOH Key Laboratory of Systems Biology of Pathogens, Institute of Pathogen Biology, Chinese Academy of Medical Sciences & Peking Union Medical College, Beijing 100730, PR China

## Abstract

*Trichophyton rubrum*, an anthropophilic and cosmopolitan fungus, is the most common agent of superficial mycoses. In this study, *T. rubrum* infection was modelled by adding human skin sections to a limited medium containing glucose and cDNA microarrays were used to monitor *T. rubrum* gene expression patterns on a global level. We observed that exposure to human skin resulted in upregulation of the expression levels of *T. rubrum* genes related to many cellular and biological processes, including transcription and translation, metabolism and secondary transport, the stress response, and signalling pathways. These results provide a reference set of *T. rubrum* genes whose expression patterns change upon infection and reveal previously unknown genes that most likely correspond to proteins that should be considered as virulence factor candidates and potential new drug targets for *T. rubrum* infection.

## Introduction

*Trichophyton rubrum* is a filamentous fungus found throughout the world that can infect human keratinized tissue (skin, nails and, rarely, hair), and is the causal agent of 80–90 % of all chronic and recurrent dermatophytoses (Costa *et al.*, 2002; Jennings *et al.*, 2002; Monod *et al.*, 2002). This pathogen, which normally causes well-characterized superficial infections, also produces skin infections in unusual parts of the body in immunosuppressed patients (Sentamilselvi *et al.*, 1998; Smith *et al.*, 2001; Squeo *et al.*, 1998). Although not normally life-threatening, dermatophyte infections are often difficult to eliminate completely and they have a 25–40 % recurrence rate (Sentamilselvi *et al.*, 1998). Additionally, resistance to antifungal drugs poses an increasing problem in clinical treatment; over the past decade, an increasing number of cases of azole- and terbinafine-resistant dermatophyte infections have been reported (Fachin *et al.*, 1996; Smith *et al.*, 2001).

The prevalence of infections caused by *T. rubrum* and its human-specific nature make it a good model for the study of human pathogenic filamentous fungi. The epidemiology, clinical case reports, strain-relatedness and drug susceptibilities of the organism are well documented (Kane *et al.*, 1997). Several studies on *T. rubrum* pathogenicity have provided evidence that genes involved in secretory activity and membrane transporters play roles in the infection process of the fungus (Fachin *et al.*, 2006; Maranhão *et al.*, 2009). Despite its limited total number of genes, a cDNA microarray analysis devised to monitor the gene expression profile of *T. rubrum* during culture in keratin–soy-protein-containing medium revealed some genes and dermatophyte-specific mechanisms involved in the putative processes related to *T. rubrum* infection (Staib *et al.*, 2010; Zaugg *et al.*, 2009). All these findings provided important information about the biological characteristics of *T. rubrum* and enhanced our understanding of its pathogenicity.

Previously, our group reported a sequencing program of over 40 000 expressed sequence tags (ESTs) derived from 10 different stages of the *T. rubrum* life cycle, which represented the first significant step towards a comprehensive description of the cellular functions involved in *T. rubrum* biology (Wang *et al.*, 2004; Yang *et al.*, 2007). In this study, we added human skin sections to a limited *T. rubrum* growth medium, and used cDNA microarray analysis to compare the transcription patterns of dermatophyte fungi in skin suspension medium and limited medium (LM). The results reveal a number of genes for which expression may be specifically regulated during infection, as well as genes related to virulence and adaptation, thereby suggesting new potential drug targets for the treatment of infections mediated by this pathogen.

## Methods

### 

#### Ethics statement.

This study was approved by the Review Board of the Institute of Pathogen Biology. The skin samples used were collected from an abdominoplasty and were removed during the normal course of surgery. This was only done on the one occasion. Written informed consent was obtained from the patient who donated the skin sample.

#### Skin sections.

The skin sections used in this study were collected from a woman who underwent an extended abdominoplasty. The normal thigh skin was incised into pieces measuring approximately 0.1–0.2 cm^2^ with full epidermal and dermal thickness, and the pieces were placed in small Petri dishes. The skin pieces were immersed in cold sterilized PBS. Whenever possible, the skin samples were used within 1 h of removal.

#### Strain and culture conditions.

The *T. rubrum* clinical isolate BMU01672 used throughout this study was obtained from a patient suffering from tinea unguium. The strain was confirmed as *T. rubrum* by morphological identification, as well as by PCR amplification and sequencing of the 18S rDNA and internal transcribed spacer regions. Strain reference samples were stored at −20 °C in the Research Centre for Medical Mycology, Beijing, PR China (Wang *et al.*, 2004).

*T. rubrum* microconidia were isolated as reported previously and adjusted to a concentration of 5–8×10^8^ conidia ml^−1^ and used as inoculum (5×10^6^ conidia ml^−1^) in the following experiments (Liu *et al.*, 2007). Incubation was at 28 °C in liquid Sabouraud medium (containing 49 g glucose, 10 g Difco Bacto Peptone in 1 l distilled water) with constant shaking (orbital, 200 r.p.m.) for 72 h to the exponential growth phase. Mycelia were harvested by filtration and washed twice with a saline solution (0.9 % NaCl), transferred into LM [glucose, 20 g l^−1^; (NH_4_)_2_HPO_4_, 6.6 g l^−1^; KH_2_PO_4_, 0.46 g l^−1^; KH_2_PO_4_·3H_2_O, 1.3 g l^−1^; MgSO_4_·7H_2_O, 0.49 g l^−1^], and incubated for 24 h. The aim of this incubation step was to eliminate the interference of the Sabouraud medium, which contained protein components that may have influenced gene expression. To analyse the gene expression of the clinical isolate under infection conditions, 2 g human skin sections were added to each of 12 flasks (250 ml), each with 80 ml LM. Another group of 12 flasks with an equal volume of LM but lacking the human skin sections was used as a control. Fungal mycelia (200 mg) were introduced into every flask and the flasks were then incubated at 28 °C with constant shaking. Three samples taken directly from the fungal mycelia were used as the 0 h samples. Subsequent samples were independently cultured, and harvested at 1, 3, 6 and 12 h.

#### RNA extraction and cDNA microarray construction.

At every time point, triplicate samples were collected from the two conditions (i.e. with and without skin). The mycelia were then ground into powder with a mortar and pestle in liquid nitrogen to facilitate cell disruption. Total RNA was isolated using the RNeasy Plant Mini kit (Qiagen) according to the manufacturer’s instructions. The RNA concentration was quantified by measurement of OD_260_. The quality of the total RNA was determined by capillary electrophoresis analysis using an Agilent 2100 Bioanalyser. Poly(A)^+^mRNA was isolated with the Oligotex mRNA Mini kit (Qiagen).

Test mRNA enriched from 50 µg total RNA was reverse transcribed into cDNA and labelled with the fluorescent dye Cy5. Additionally, reference genomic DNA (5 µg) isolated at the hyphal stage was labelled with Cy3 (Liu *et al.*, 2007).

#### cDNA microarray preparation and hybridization.

The microarrays used in this study contained a total of 10 000 spots, including 8997 clones in the form of PCR products, which represented ~80 % of the genes of *T. rubrum* and 1003 controls. The PCR fragments used to print the microarray chip were amplified from our EST libraries with T7 and SP6 universal primers. Microarray preparation and hybridization were performed as described previously (Liu *et al.*, 2007).

#### Data acquisition and microarray data analysis.

After hybridization, the slides were scanned using a GenePix 4000A microarray scanner (Axon Instruments) and the acquired microarray images were analysed using GenePix Pro 6.0 software. The data were filtered for spots fitting all of the following features: spot diameter ≥80 µm, % B (532 or 635)+2SD >55, SNR635 (or 532) ≥3. The filtered data were then normalized (the ratio of medians of all features equal to 1) by the GenePix Pro 6.0 software. The datasets were further normalized with a total intensity and Lowess normalization using TIGR midas V2.19. After normalization, the expression data of samples cultured for 0, 1, 3, 6 and 12 h in suspension medium were subjected to one-way ANOVA (*P*<0.01) using TIGR MultiExperiment Viewer (MeV) software to identify the genes whose expression levels were altered significantly during the culturing process in skin suspension medium. To identify the genes whose expression levels were altered dramatically in response to human skin induction rather than in a time-dependent manner, ANOVA was also used to compare the differences in the expression pattern between *T. rubrum* cultured in suspension medium and LM (*P*<0.01). Finally, the changes in the expression levels of each significant gene were clustered using TIGR MultiExperiment Viewer (MeV) software and the *k*-means clustering (KMC) method.

#### Microarray data accession number.

Microarray experimental design, hybridizations and data processing in this study complied with the Minimum Information About Microarray Experiment guidelines. All of the EST sequences used for cDNA microarray construction have been submitted to GenBank under accession numbers DW405580–DW407270 and DW678211–DW711189, and the GenBank accession numbers of ESTs corresponding to the positions on the cDNA microarray have been deposited in the Gene Expression Omnibus repository under the accession number GPL7240. The microarray data have been deposited in the Gene Expression Omnibus repository under the accession number GSE15532. Detailed information about the EST contig assemblies and EST annotations can be obtained from our *T. rubrum* database (http://www.mgc.ac.cn/TrED/).

#### Validation of microarray data by real-time reverse transcription (RT)-PCR.

To verify the microarray results, the relative expression levels of 15 genes in the study were estimated by quantitative real-time RT-PCR. Aliquots of the RNA preparations from samples at each of the time points used in the microarray experiments were saved for quantitative real-time RT-PCR. Gene-specific primers were designed for the genes and the 18S rRNA using Primer Express software (Applied Biosystems) (Table S1, available in the online Supplementary Material). PCRs were performed on an Applied Biosystems GeneAmp7000 sequence detection system. Changes in the fluorescence of SYBR Green I dye were monitored by GeneAmp7000 software and the threshold cycle (*C*_t_) above the background for each reaction was calculated. The *C*_t_ value of 18S rRNA was subtracted from that of the gene of interest to obtain a Δ*C*_t_ value. The Δ*C*_t_ value of an arbitrary calibrator (e.g. an untreated sample) was subtracted from the Δ*C*_t_ value of each sample to obtain a ΔΔ*C*_t_ value. The gene expression level relative to the calibrator was expressed as 2^−ΔΔ^*^Ct^*.

## Results

### Highly and differentially expressed genes in the *T. rubrum* infectious model

**Fig. 1.**
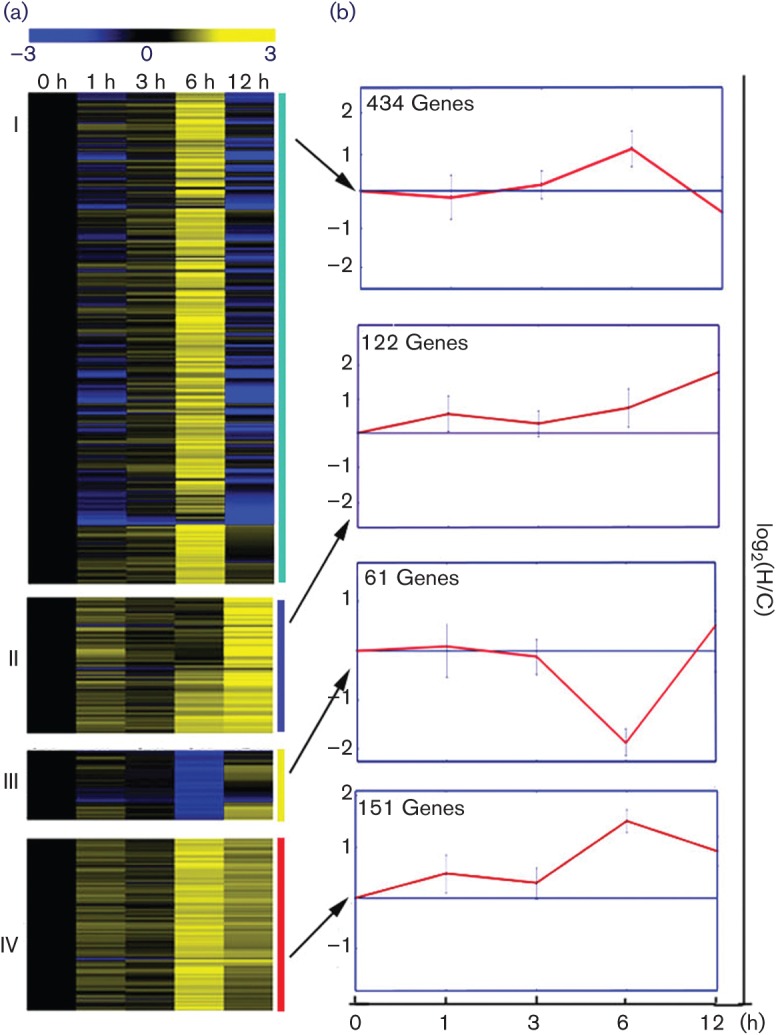
KMC clustering of microarray data and identification of genes with similar transcriptional profiles. (a) A total of 768 genes were clustered on the basis of their expression profiles when cultured with human skin sections relative to their expression profiles when cultured in LM across five time points. Each gene belonged to one of the four KMC clusters. Each gene’s expression values were standardized to have a median of 0 and an sd of 1 across the time points from 0 to 12 h. The lighter colour in the cluster dendrogram is correlated with a higher expression level. (b) Mean expression profiles of the genes within each cluster. To obtain each profile, a sum of the expression values across the five time points for each gene was standardized to 1. Next, the time-course values for all genes in each cluster were summed and the summed value for the five time points for each cluster was scaled to 1. log2(H/C): for each gene in KMC clusters, log2(H/C) refers to log2 (expression level cultured in human skin sections/expression level cultured in LM).

To assess gene expression patterns during the infection process, the mycelia of a *T. rubrum* isolate were grown in a human skin suspension medium and equal amounts of mycelia were introduced into LM as a control. During the 12 h culture process, the growth of the mycelia in these two media showed no obvious differences. The skin sections in the medium were also not consumed visibly. Changes in gene expression were monitored from 0 to 12 h using a cDNA microarray. A total of 2452 genes were selected based on the significant changes in expression by ANOVA (*P*<0.01) during this period. Using ANOVA (*P*<0.01) by TIGR MeV software (Saeed *et al.*, 2003), the expression patterns of the 2452 selected genes were compared with the corresponding data from the control LM culture. Of these 2452 genes, the expression changes of 1258 genes were confirmed as due mainly to human skin induction, rather than simply being time-dependent. Of these 1258 genes, 768 demonstrated changes in expression levels exceeding a twofold difference and were used for further analysis to identify genes whose expression levels were altered dramatically in response to human skin induction (for detailed results, see Table S2). To verify the microarray results, the relative expression levels of 15 genes were estimated by quantitative real-time RT-PCR. The results showed a strong positive correlation between the two techniques (Table 1). These 768 genes were then subjected to KMC analysis using TIGR MeV software to evaluate the relative expression pattern of *T. rubrum* cultured in skin suspension medium (Saeed *et al.*, 2003). Each of these 768 genes belonged to one of four KMC clusters (Fig. 1).

**Table 1.** Relative fold change for 15 genes listed determined by quantitative real-time RT-PCR and microarray hybridization results Column R shows the fold change after adding human skin sections relative to LM, determined by quantitative real-time RT-PCR. Column M shows the fold change after adding human skin sections relative to LM, determined by the microarray hybridization results. The correlation coefficient (*r*) for these two technologies was calculated using SPSS 13.0 software.

ESTs	Cluster	0 h		1 h		3 h		6 h		12 h		*r*
		R	M	R	M	R	M	R	M	R	M
DW685106	I	1	1	1.0324	0.5678	0.52159	1.07093	4.53154	2.91846	1.2781	0.26321	0.89
EL788295	IV	1	1	1.20497	1.40535	0.92916	1.27826	7.12554	3.01009	1.18509	1.52157	0.976
DW680891	I	1	1	1.31859	1.19658	0.82817	1.60904	2.16145	3.0985	0.46041	0.77202	0.884
DW680904	I	1	1	0.993781	1.103348	0.56331	1.183078	2.540302	2.775284	1.585568	1.464977	0.929
DW685928	I	1	1	0.959264	0.706954	1.624505	1.261124	1.58447	2.169198	1.024557	0.673213	0.801
DW680574	I	1	1	0.984866	1.127878	0.628507	1.221062	2.760635	2.466178	1.071031	0.770112	0.906
DW706077	IV	1	1	1.449947	1.466285	0.842063	1.236475	3.874473	3.165263	0.951978	1.872483	0.919
DW678823	I	1	1	0.681601	1.245822	1.701727	1.2606	3.805273	2.114076	1.063633	1.089313	0.946
DW679448	IV	1	1	1.330529	1.423327	0.541488	1.290017	2.270484	2.312821	1.138394	1.52867	0.875
DW680706	II	1	1	0.986233	1.246938	0.315126	1.022999	1.91587	1.752762	2.040609	2.356341	0.891
EL786224	I	1	1	3.228804	0.708054	0.571173	1.022053	5.490738	1.709587	1.313121	0.424985	0.646
EL792939	I	1	1	2.363623	0.774499	0.566049	0.986895	5.43018	2.6246	2.16595	0.617136	0.831
DW678242	I	1	1	1.139183	0.831055	0.384752	1.277211	5.333195	2.962537	2.475981	0.462158	0.765
EL785855	I	1	1	1.504204	0.706083	0.428094	0.998018	3.292081	2.441484	1.135242	1.038479	0.871
DW703795	I	1	1	1.201636	0.568963	0.741234	1.054972	2.171964	1.680151	0.455019	0.236967	0.815

Clusters I and IV contained 434 and 151 of the 768 genes, respectively. Transcripts of genes in these two clusters were induced to increase from the zero time point, with a peak expression at 6 h. Genes in cluster IV maintained high expression levels at each time point examined, while the expression level of the cluster I genes declined rapidly after 6 h. There were 122 genes assigned to cluster II, whose mRNA accumulated throughout the skin induction process. The transcript levels of the 61 genes in cluster III had low expression levels throughout the process and they exhibited their lowest expression at 6 h.

### Metabolic changes induced by human skin sections

The genes that were induced by the addition of human skin sections are listed in Table S1 and the distribution of the functional categories is shown in Table 2. Compared with the control LM culture, the addition of human skin sections induced prominent changes in metabolism in *T. rubrum*. In our analysis, ~231 of the 768 genes functioned in the ‘metabolism’ category.

**Table 2.** Functional annotation and cluster distribution of genes induced by human skin sections Individual genes can take part in multiple biological processes. The detailed results are provided in Table S2. Detailed annotation and function characterization of the *T. rubrum* ESTs is also depicted in Table S1 and our *T. rubrum* database (http://www.mgc.ac.cn/TrED/).

Biological process	Cluster I	Cluster II	Cluster III	Cluster IV
Metabolism				
Carbohydrate metabolic process and energy pathways	48	11	3	15
Protein metabolic process, proteolysis	22	2	0	13
Amino acid metabolic process	35	6	1	6
Lipid metabolic process	8	3	1	4
Nucleic acid metabolic process	10	0	0	5
Secondary metabolites	6	3	2	4
Cell organization and biogenesis	9	3	1	0
Cell division, reproduction	12	4		10
Transcription	17	10	2	5
Translation and protein biosynthesis	73	2	0	9
Response	5	4	0	5
Regulation of biological process	7	0	0	1
Transport	29	17	0	13
Other	25	13	1	11
Biological process unknown	132	57	50	52

In the metabolism category, 59 genes were involved in primary carbohydrate metabolism, including glycolysis, the tricarboxylic acid cycle and oxidative phosphorylation (Table S3). Most of these genes were in clusters I and IV, which exhibited induced expression from the zero time point and reached peak expression at 6 h.

Three genes involved in the chitin metabolic process (DW695173, DW697427 and EL793695) and DW685106, which encodes the cell wall enzyme 1,3-β-glucanosyltransferase, were induced upon addition of human skin sections.

Thirty-seven genes relative to protein metabolism were induced during incubation (Table S4), nine of which were potential secreted proteases or peptidases, such as aspartyl protease, serine-type peptidase, metallopeptidase and prolidase pepP, which are likely involved in proteolysis and related to pathogenicity (Krappmann & Braus, 2005; Monod *et al.*, 2002; Naglik *et al.*, 2003).

Twenty-four other genes encoding components of the proteasome and ubiquitin-conjugating enzymes E2 and E3 were also identified (Table S4).

Forty-eight genes were identified that functioned in amino acid metabolic pathways, including those for arginine, glutamine, proline, lysine, methionine, threonine, histidine and glutamate, as well as pathways related to aromatic amino acid biosynthesis, glutamate metabolism, methionine metabolism and arginine metabolism (Table S5). Apart from one gene that belonged to cluster III, the other 47 genes belonged to clusters I, II and IV, which were overexpressed during the culturing process. In our analysis, two putative genes, DW678242 and DW680904, encoding homocitrate synthase and α-aminoadipate reductase involved in lysine biosynthesis, were induced throughout the infection process. As α-aminoadipate reductase is a key enzyme in the branched pathway for lysine and β-lactam biosynthesis of filamentous fungi, and impaired lysine biosynthesis can severely attenuate virulence in Aspergillus *nidulans* and Aspergillus *fumigatus* (Liebmann *et al.*, 2004; Tang *et al.*, 1994), our results suggest that lysine biosynthesis is related to the process of *T. rubrum* infection as well.

Methionine production is also essential for protein biosynthesis in micro-organisms. Our data indicate that the gene EL785855, which encodes aspartate semialdehyde dehydrogenase, was upregulated; this enzyme functions at the first branch point in the biosynthetic pathway through which bacteria, fungi and higher plants use aspartate to synthesize certain amino acids, including lysine and methionine.

Secondary metabolites from fungi are usually active as mycotoxins and virulence factors that may be involved in the pathogenic development of fungal infections (Bennett & Klich, 2003; Rementeria *et al.*, 2005). Two genes, DW680904 and DW680891, involved in ergot alkaloid biosynthesis, encoding l-aminoadipate-semialdehyde dehydrogenase and dimethylallyl tryptophan synthase, were observed in cluster I. The ergot alkaloids are a family of indole-derived mycotoxins that have a variety of important biological activities (Coyle & Panaccione, 2005; Tudzynski *et al.*, 2001). Knocking out the gene encoding dimethylallyl tryptophan synthase resulted in the loss of all known ergot alkaloids in *Aspergillus fumigatus* and clavicipitaceous fungi, which indicated that dimethylallyl tryptophan synthase controls the determinant step in the ergot alkaloid pathway (Coyle & Panaccione, 2005).

### Changes in the expression of genes related to the cell cycle and translation

After the addition of human skin sections to LM, the expression level of 60 genes involved in the cell cycle and in basic genetic information flow machinery, including DNA replication, repair, recombination, transcription and RNA processing, were changed significantly (Table S6). Among these genes, 14 of 19 genes related to the structure and assembly of chromatin, chromosome segregation and cell cycle control were induced. The expression level of genes related to transcription was also altered after the addition of human skin sections. Thirty-two genes were assigned to ‘transcription’ and 27 of them, grouped into clusters I or IV, were induced.

Translation was also changed notably in response to the addition of human skin sections. In the infection process, the expression of 84 genes encoding proteins that functioned in ribosomal components or protein synthesis was modified (Table S7). These 84 genes were suppressed at the beginning, induced at 3 h and reached the highest expression levels at 6 h. Thirty-six genes were ribosomal components and 35 other genes belonged to cluster I. Forty-eight of the other 49 genes, including those encoding translation initiation factor 2, 3 and 4, translation elongation factor Tu, peptide chain release factor 1, tRNA synthetases, and factors involved in protein folding, were assigned to clusters I and IV, and were induced by human skin sections.

### Changes in transport gene expression upon the addition of human skin sections

The expression of 60 genes related to transport was modified in response to the addition of human skin sections (Table S8). Thirty of these genes were assigned to cluster I, 17 of them were assigned to cluster II and 13 of them were assigned to cluster IV. Of these 60 genes, 12 upregulated genes were related to vesicle-mediated autophagy and exocytosis. Additionally, 12 genes encoding major facilitator superfamily transporter proteins, two genes encoding ATP-binding cassette transporters, and three genes encoding proteins involved in iron and copper transport were also induced. These transporter proteins have remarkably broad substrate specificity, and are able to transport a wide variety of natural and synthetic toxic products of either endogenous or exogenous origin (Pao *et al.*, 1998; Stergiopoulos *et al.*, 2002). When functioning as virulence factors, these membrane transporters are required to overcome host defence mechanisms and are important for *T. rubrum* pathogenicity (Maranhão *et al.*, 2009).

### Changes in stress response genes upon the addition of human skin sections

We observed changes in the expression of 14 genes encoding proteins with a known or putative role in the response to temperature shift or oxidative stress (Table S9). These proteins included two molecular co-chaperones associated with cellular responses to temperature shifts, HSP70 (DW681773) and HSP90 (DW693707); one superoxide dismutase (EL788295), and three novel cytochrome *c* oxidases associated with antioxidant function (DW686878, DW700639 and DW696907). Three homologues of genes encoding secondary antioxidant enzymes belonging to the glutathione *S*-transferase family (DW679047, DW679402 and DW406127) were also identified.

The signal transduction pathways are conserved and mediate adaptive changes in many fungi, including cellular development and morphogenesis (d'Enfert, 1997; Fillinger *et al.*, 2002; Liebmann *et al.*, 2004). In pathogenic micro-organisms, these signalling pathways may also be used to regulate the determinants of virulence and host infection (Hamilton & Holdom, 1999; Rementeria *et al.*, 2005). We identified seven genes involved in mitogen-activated protein kinase signalling and Rab/RAS small GTPase-mediated signalling pathways, and we assigned them to clusters I and IV (Table S10). Some of these genes encoded proteins in these signalling pathways that have been associated with virulence in *Aspergillus fumigatus* and *Candida albicans* (Hogan *et al.*, 1996; Rementeria *et al.*, 2005). The role of these signalling pathways in *T. rubrum* infection requires further study.

## Discussion

In this study, we modelled a *T. rubrum* infection by adding human skin sections to LM containing glucose to monitor global *T. rubrum* gene expression patterns using cDNA microarrays. Our results reflect the adaptation that results from the interaction of *T. rubrum* with its human host. In host–pathogen interactions, the gene expression of the pathogen is modulated by signals from the host and understanding the resulting expression patterns may provide insight into the disease mechanism. The transcription profile monitoring revealed that the expression levels of genes related to many cellular and biological processes of *T. rubrum* were induced by the addition of human skin sections, including transcription and translation, metabolism and secondary transport, stress response, and signalling pathways. The cell cycle is the basic cellular process involved in cell division, polar construction, morphogenesis and adaptation to different environments. We observed that a number of genes associated with cell cycle events, including the assembly of chromatin, chromosome segregation and cell cycle control, were upregulated. The transcription of genes encoding certain ribosomal subunits and translation factors was also upregulated highly after the human skin sections were added. These results suggest that the expression of many genes changes dramatically as *T. rubrum* adapts to the presence of human skin sections.

The secretion of proteolytic enzymes by dermatophytes is a key factor in the invasion and utilization of the stratum corneum of the host (Grumbt *et al.*, 2011; Maranhão *et al.*, 2009; Monod, 2008). Recently, seven dermatophyte genomes have been completely sequenced and all of them were found to encode high numbers of proteases compared with other non-dermatophytic fungi (Achterman & White, 2012; Burmester *et al.*, 2011; Martinez *et al.*, 2012). Secretion of several *T. rubrum* proteolytic enzymes has been observed and is thought to be involved in infection (Apodaca & McKerrow, 1989; Leng *et al.*, 2009; Maranhão *et al.*, 2007; Monod, 2008). Using a *T. rubrum* cDNA microarray, pathogenic dermatophyte *Arthroderma benhamiae* and the molecular basis of keratin degradation in *T. rubrum* indicated that numerous genes encoding secreted proteases were induced in these two fungi (Staib *et al.*, 2010; Zaugg *et al.*, 2009). In the present study, certain genes involved in protein metabolism were induced, including several types of potential secreted proteases. Most genes involved in multiple protein transport pathways, autophagy and exocytosis were also upregulated. These results may improve our understanding of the pathogen and its ability to use extracellular enzymes to degrade the structural barriers of the host. We also observed the induction of *T. rubrum* genes involved in the biosynthesis of various amino acids and in amino acid metabolism. In these pathways, lysine and methionine biosynthesis have been identified as contributors to virulence in many fungal pathogens (Liebmann *et al.*, 2004; Tang *et al.*, 1994), and they may also have a role in the *T. rubrum* infection process. These pathways are non-existent or they function differently in animals and humans. We hypothesize that certain genes that encode enzymes involved in the key steps of these pathways are promising antifungal drug targets. Some genes and pathways involved in secondary metabolism, the response to stress and the transport of toxic components were also induced after the addition of human skin sections. These genes and related pathways may be involved in virulence and determining the sensitivity to fungicides and other antimycotic agents.

*T. rubrum* is a human-specific pathogenic fungus. Due to the lack of an animal model to study biological processes involved in *T. rubrum* infection, the functional analysis of *T. rubrum* described here will provide important information regarding its pathogenicity and virulence.
